# Assessment of the main plasma parameters included in a metabolic profile of dairy cow based on Fourier Transform mid-infrared spectroscopy: preliminary results

**DOI:** 10.1186/s12917-015-0621-4

**Published:** 2016-01-06

**Authors:** Luigi Calamari, Annarita Ferrari, Andrea Minuti, Erminio Trevisi

**Affiliations:** Istituto di Zootecnica, Facoltà di Scienze Agrarie, Alimentari e Ambientali, Università Cattolica del Sacro Cuore, Piacenza, 29122 Italy

**Keywords:** Dairy cows, Metabolic profile, FT-MIR spectroscopy, Infrared model prediction

## Abstract

**Background:**

Although a metabolic profile represents a valid tool utilized in dairy herds to determine abnormalities in blood chemistry related to an increased risk of production diseases, there are no studies on application of Fourier Transform mid-infrared (FT-MIR) spectroscopy. This study assesses the potential application of FT-MIR to analyze the main blood biochemical parameters included in the metabolic profile of dairy cows. Infrared transmission spectra were acquired for 35 plasma samples (two replicates on each sample) of Italian Friesian dairy cows (14 primiparous and 21 pluriparous), all without clinical events, and at different stages of lactation, although mainly in the transition phase. Each sample was also analyzed independently using accepted reference clinical chemical methods and these results were used as calibrating values to perform predictive models by PLS method using cross validation.

**Results:**

Measured blood parameters concentrations were all within the reference ranges reported for healthy dairy cows. The number of extracted factors with the PLS procedure for each prediction model ranged between 3 and 7. The coefficient of determination (R^2^) of the prediction models ranged between 0.1 to values close to 1. R^2^ values greater than 0.9 were observed for the prediction models of total cholesterol, total protein, globulin, and albumin; values between 0.75 and 0.9 were observed for urea, NEFA, and total bilirubin, while values of R^2^ lower than 0.6 were observed for all minerals and for enzyme activity. The range error ratio (RER) and prediction to deviation (RPD) ranged from 5.1 to 43.8 and from 1 to 13.8 for RER and RPD, respectively. Values of RPD greater than 5 were observed for total cholesterol, total protein, albumin, and globulin. RPD ranged between 2 and 5 for the prediction models of urea, NEFA, and total bilirubin, while RPD and RER were low for minerals and enzyme activities.

**Conclusions:**

Although the results of this study require further validation, the use of FT-MIR spectroscopy was possible and provides fairly accurate measurement of various parameters of great importance in the evaluation of the metabolic and inflammatory status in dairy cows.

## Background

Blood biochemistry is mainly used in diagnostic investigation to provide supportive evidence of a suspected diagnosis, as a prognostic indicator, or for monitoring the progress of disease in animals that are being treated. Payne et al. [1] proposed that blood tests could be included in a metabolic profile of dairy herds to indicate an inability to maintain homeostasis, with the aim to determine abnormalities in some blood parameters and, the signaling of an increase in the risk of production diseases. Recently, Clarke and Haselden [2] extended the meaning of metabolic profiling and include the measurement of any parameter in animal fluids that is able to reflect a dynamic response to genetic modification, and physiological, pathophysiological and developmental stimuli. Although substrate and analytical methods are similar in evaluating disease diagnosis and metabolic profiling, there are different approaches to sampling and interpretation. To diagnose disease, a small population of clinically affected animals are selected for the blood analysis. Diagnosis of disease is based on changes in one or more blood analytes. Conversely, for a proper assessment of the metabolic profile blood, samples are collected from clinically healthy animals within certain well-defined physiological phases [3]. The metabolic profile is then used to evaluate disease risk in contrast to disease diagnosis. When used in association with animal, diet and management assessments, a metabolic profile can be a useful tool for prediction of periparturient problems and infertility, to diagnose metabolic diseases, to assess nutritional status [4–6], stress conditions [7, 8], and to determine welfare condition [9, 10].

Because a metabolic profile considers clinically healthy animals, the variability of analytes is lower than the variability in blood biochemistry for diagnosis of diseases. As such, a metabolic profile requires high precision and an accurate analytical method.

The blood tests included in a metabolic profile are in generally analyzed by using an automated or semi-automated analyzer. However, some critical aspects can restrict its use, including the cost of analytical testing and in some cases the long waiting time between the sampling and return of analysis results. Human studies have demonstrated the potential of infrared spectroscopic methods for reliable clinical chemistry [11, 12], as well as clinical applications and diseases diagnosis [13].

Applications of infrared spectroscopy as an analytical tool in different areas of animal production are receiving growing interest and attention. This technology is used to assess feed composition and digestibility, traceability assessment, and for determination of the main composition of animal products (meat, milk, fish, cheese, eggs) [14]. For more information see the review paper by Berzaghi and Riovanto [14]. Infrared spectroscopy has several advantages over other analytical techniques: rapidity of analysis, no use of chemicals, minimal or no sample preparation, and easy applicability in different work environments (on/in/at line applications) [11, 15].

To the best of our knowledge no reports have investigated the possibility of using infrared spectroscopy to analyze a metabolic profile in dairy cows. Considering the results obtained in human studies that demonstrate the potential application of infrared spectroscopy in this field, the objective of the present research was to test the feasibility of developing prediction models of the main blood biochemical parameters included in a metabolic profile for dairy cows using MIR spectrometry.

## Methods

### Animal and management conditions

The research protocol and the animal care were in accordance with the Directive 2010/63/EU (European Union, 2010) on the protection of animals used for scientific purposes. The cows recruited in this trial were involved in Research Projects authorized by Italian Ministry of Health and approved by the ethics committee (Commissione per la valutazione etica di sperimentazioni animali e di correttezza della gestione dell’animal care) prot. N. 25906/13 of 22 Nov 2013.

The Italian Friesian dairy cows involved in this study were raised in the experimental free stall barns of the Università Cattolica del Sacro Cuore (Piacenza, Italy). Dry cows and lactating cows were kept in two adjoining pens. The cows were moved from a far-off pen to a late pregnancy pen one month before the expected calving day and the heifers were moved two months before their expected calving day. After calving the cows were housed in the pen for lactating cows. Fresh potable water was available *ad libitum* within each pen. Cows were fed TMR once daily for *ad libitum* DMI, at 0830 h and 1030 h for lactating and dry cows, respectively. Diets were formulated to meet requirements according to NRC system (2001).

The trial involved 35 Italian Friesian dairy cows (14 primiparous and 21 pluriparous) of good genetic merit, in the last three months of pregnancy and till 280 days in milk. The 40 % of cows (14 animals) were in the transition period (from 21 days before calving till 21 days after calving).

### Blood sampling and analyses with reference methods

Blood samples were collected from all cows all at one time. The samples were collected in the morning, before the feed distribution, by venipuncture from the jugular vein, using 10-mL Li-heparin treated tubes (Vacuette, containing 18 IU of Li-heparin mL^−1^, Kremsmünster, Austria). Samples were immediately cooled in an ice-water bath after collection.

The blood was centrifuged (3500 × g for 16 min at 4 °C) and the plasma obtained was separated into two aliquots: the first fraction was immediately used to collect the infrared spectra; the second one was stocked at −20 °C until metabolites analysis and the results were used as calibration values.

Plasma metabolites used as calibrating values were analyzed at 37 °C by an automated clinical analyzer (ILAB 600, Instrumentation Laboratory, Lexington, MA), using the methodology showed in Table 1. Commercial kits were used to measure glucose, total cholesterol, urea, calcium, inorganic phosphorus, magnesium, total protein, albumin, total bilirubin, and creatinine (Instrumentation Laboratory SpA, Werfen, Monza, Milan, Italy), NEFA and zinc (Wako, Chemicals GmbH, Neuss, Germany), and β-OH-butyric acid (BHBA, kit Ranbut, Randox Laboratories Limited, Crumlin, County Antrim, United Kingdom Randox, UK). A Trinder end point [Glucose oxidase (GOD)/Peroxidase (POD)] was used to measure glucose. Total cholesterol (cholesterol and cholesterol esters) was also measured using Trinder end point [Cholesterol oxidase (CHOD)/Peroxidase (POD)], after a hydrolysis of cholesterol esters to free cholesterol. Urea was measured with end point method using the couple urease glutamate dehydrogenase (GLDH) enzyme system. Colorimetric methodology based on the reaction of calcium with o-cresolphthalein complexone was used to measure Ca with end point method. Inorganic phosphorus was measured with end point UV method, based on the reaction between phosphate ions in an acidic medium with ammonium molybdate to form a phosphomolybdate complex. The magnesium determination was based on the reaction of magnesium with Xylidyl Blue (as chelator) at alkaline pH, which yields a purple colored complex. Total protein were measured with the modified biuret methodology, based on the reaction of peptide bonds with Cu^++^ ions in alkaline solution to form a colored complex. Albumin was measured with an end point colorimetric method, based on the binding between albumin with green bromocresol resulting in a spectral change of the dye from yellow to green. Total bilirubin was determined with an end point analysis using modified Jendrassik-Grof method, based on the reaction between total bilirubin with diazotized sulfanilic acid in presence of lithium dodecylsulfate to form azobilirubin. Creatinine was analyzed with an end point colorimetric method, based on the reaction of creatinine with picric acid under alkaline conditions. NEFA were determined with an Trinder end point [Acyl coenzyme A oxidase (ACOD)/Peroxidase (POD)] assay, after the acylation of coenzyme A by NEFA contained in the sample. BHBA was measured with a kinetic UV method, based on the oxidation od D-3 hydroxybutyrate to acetoacetate by 3-Hydroxybutyrate dehydrogenase. Electrolytes, Na, K, and Cl, were measured using a potentiometric system, with specific electrodes. Kinetic analysis was adopted to determine the activity of enzymes: alkaline phosphatase (AP, EC 3.1.3.1), aspartate aminotransferase (AST, EC 2.6.1.1), γ-glutamyltransferase (GGT, EC 2.3.2.2) using Instrumentation Laboratory kits (Instrumentation Laboratory SpA, Werfen, Monza, Milan, Italy). Ceruloplasmin was measured using the method described by Sunderman and Nomoto [16], adapted to ILAB 600 condition. The method is based on measurement of p-phenylenediamine dihydrochloride oxidation by the oxidase activity of ceruloplasmin. Finally, haptoglobin was measured using the method described by Skinner et al. [17] and Owen et al. [18] and adapted to ILAB 600 condition. The method is based on peroxidase activity of methaemoglobin-haptoglobin complex measured by the rate of oxidation of guaiacol (hydrogen donor) in presence of hydrogen peroxide (oxidizing substrate).

**Table 1:** Methodologies used to measure the plasma parameters with reference methods

Parameter	Methodology	Wavelength (nm)	CV^a^
Glucose	Endpoint	510	1.50
Total cholesterol	Endpoint	510	2.10
NEFA^b^	Endpoint	546	1.50
BHBA^c^	Endpoint	340	5.25
Urea	Endpoint	340	1.20
Creatinine	Endpoint	510	5.40
Ca	Endpoint	570	1.40
Inorganig P	Endpoint	340	2.00
Mg	Rate	340	1.40
Na	ISE device^g^		0.90
K	ISE device^g^		1.30
Cl	ISE device_g_		1.50
Zn	Endpoint	546	
Ceruloplasmin	Endpoint	546	3.48
Total protein	Endpoint	546	1.20
Albumin	Endpoint	600	1.80
Total bilirubin	Endpoint	546	6.70
Haptoglobin	Endpoint	450	13.54
AST^d^	Rate	340	2.10
GGT^e^	Rate	405	3.72
AP^f^	Rate	405	1.70

^a^Calculared on the results obtained between runs according to the National Committee for Clinical Laboratory Standards (Document EP3-T: Guidelines for Manufacturers for Establishing

Performance Claims for Clinical Chemistry Methods, Replication Experiment

Evaluation”, Villanova, PA, 1982.)

^b^Non esterified fatty acids;

^c^β-OH-butyric acid;

^d^Aspartate amino transferase

^e^γ-glutamyl transferase

^f^Alkaline phosphatase

^g^Ion selective electrodes

### FT-MIR spectroscopy

FT-MIR measurements were performed with a MilkoScan FT 120 (Foss Electric, Hillerød, Denmark). FT-MIR spectra were collected (two replicates on each sample of the calibration datasets, and one replicate on each sample of the validation dataset) as % transmittance in the range of 5012–926 cm^−1^. The transmittance spectrum measured on each sample was standardized using the FT-MIR Equalizer sample (Foss Electric, Hillerød, Denmark), in order to use the same calibration for several instruments. The instrument was standardized with the equalizer sample before reading the spectra of the samples collected from each farm.

In order to not use areas of the spectra that introduce noise to the calibration, only the following areas were used to develop the prediction model: 2971.7 to 2432.4 cm^−1^, 2272.4 to 1716.8 cm^−1^, and 1543.2 to 964.5 cm^−1^. The two areas 3626.5 to 2970.7 cm^−1^ and 1716.8 to 1543.2 cm^−1^, corresponding to water absorption areas, were not used to calibrate plasma parameters. The 5012.0 to 3626.5 cm^−1^ area was excluded as this contains very little interesting information (Foss Electric, 2002).

### Data processing

All analyses were performed using the statistical software package SAS 9.2 (SAS Inst. Inc., Cary, NC). Data were tested for non-normality by the Shapiro test. In case of non-normality, parameters were normalized by log or exponential transformation. Transformations were performed for total bilirubin, haptoglobin, AST, GGT, AP, NEFA, and BHBA.

The infra-red data were processed using the SAS statistical package. In order to suppress side effects from water absorption [19], before statistical analysis the transmittance data of each area was referred to a reference value, dividing the %T of each area by the %T of the reference area (1492 to 1481 cm^−1^). The absorbance was then calculated (−log10 of the corrected %T) for each area.

Partial least-squares (PLS) analysis was used to process the data by SAS. The selection of the optimal number of the extracted PLS factors for the prediction model of each plasma variable was obtained using cross validation to prevent overfitting. The predictors and the responses were centered and scaled to have a mean of 0 and a standard deviation of 1. Centering the predictors and the response ensures that the criterion for choosing successive factors is based on how much variation they explain, in either the predictors or the responses or both. Scaling serves to place all predictors and responses on an equal footing relative to their variation in the data. The accuracy of the predictive ability of the model is assessed by cross-validation, by means of the root mean square error of cross validation (RMSECV). All prediction residuals were then combined to compute the predicted residual of sum of squares (PRESS) [20]. A statistical model comparison [21] was applied to test whether the differences between the cross-validated residuals from models with different number of extracted factors were significant. The chosen prediction model was based on the results obtained with the cross validation, and the number of factors chosen was the fewest with residuals that were not significantly larger than the residuals of a model with minimum PRESS.

The accuracy of the calibration was evaluated considering the coefficients of determination R^2^ for predicted versus measured values in cross-validation; the ratio of the range of each variable in the data was set to its standard deviation of prediction errors; and the ratio between the standard deviation of a variable in the data set (SD) by its standard deviation of prediction errors. Values for R^2^ between 0.66 and 0.81 indicate approximate quantitative predictions, whereas, a value for R^2^ between 0.82 and 0.90 is adequate for a good prediction. Calibration models having a value for R^2^ above 0.91 are considered to be excellent [22]. The practical utility of the calibration was also evaluated by using the range error ratio (RER). This ratio was calculated by dividing the range of each variable by its standard deviation of prediction errors [23]. Values of RER below 3 indicate that a model has little practical utility; RER values between 3 and 10 are limited to good practical utility and RER values above 10 indicate models of high utility value [23]. Because RER could be susceptible to the presence of extreme values at both ends of the range, the ratio of the SD to the standard deviation of prediction errors, called the ratio of prediction to deviation (RPD), was also considered. Based on the RPD value, five levels of prediction accuracy according to Saeys et al. [24] were considered. A value for the RPD below 1.5 indicates that the calibration is not usable. Values for RPD between 1.5 and 2.0 reveal a possibility to distinguish between low and high values, while a value between 2.0 and 2.5 allows for approximate quantitative predictions. For values between 2.5 and 3.0, and above 3.0, the prediction is classified as good and excellent, respectively.

Repeatability (S_r_) was obtained as the standard deviation of the results obtained with the prediction models applied to the two replicates and infrared spectra of each sample. Repeatability relative standard deviation (RSD_r_) was also calculated.

## Results

Table 2 presents descriptive statistics. The blood parameters with the lowest variability were the electrolytes. CV was 2.4, 4.9, and 2.3 % for Na, K, and Cl, respectively. Plasma Ca was characterized by low variability (CV of 4.7 %). Greater variability was found for Mg (9.8 %), inorganic P (19.34 %) and Zn (23.7 %).

**Table 2:** Descriptive statistic (35 plasma samples)

Item	Mean	SD	Min	Max	Median	SK^a^	KU^b^
Glucose, mmol L^−1^	4.02	0.67	1.66	6.20	3.95	−0.13	5.64
Total cholesterol, mmol L^−1^	3.36	1.59	0.80	5.87	3.18	0.05	−1.57
NEFA^c^, mmol L^−1^	0.33	0.29	0.06	1.18	0.20	1.06	0.30
NEFA^c^, ln(mmol L^−1^)	−1.52	0.94	−2.83	0.16	−1.61	0.17	−1.49
BHBA^d^, mmol L^−1^	0.49	0.13	0.28	0.85	0.47	1.00	0.78
BHBA^d^, ln(mmol L^−1^)	−0.74	0.25	−1.28	−0.16	−0.76	0.34	0.06
Urea, mmol L^−1^	4.21	1.42	1.50	7.52	4.02	0.21	−0.61
Creatinine, μmol L^−1^	99.54	10.67	83.62	125.89	97.57	0.47	−0.51
Ca, mmol L^−1^	2.58	0.12	2.36	2.86	2.61	0.33	−0.16
Inorganig P, mmol L^−1^	1.81	0.35	1.19	2.89	1.74	1.09	1.36
Mg, mmol L^−1^	1.02	0.10	0.57	1.12	1.04	−2.97	9.99
Na, mmol L^−1^	144.20	3.44	138.78	151.78	143.81	0.38	−0.68
K, mmol L^−1^	4.07	0.20	3.59	4.57	4.08	−0.09	0.20
Cl, mmol L^−1^	107.51	2.48	102.27	112.98	107.46	−0.09	−0.19
Zn, μmol L^−1^	11.43	2.71	5.17	16.19	11.40	−0.48	−0.19
Ceruloplasmin, μmol L^−1^	3.24	1.02	1.99	6.63	3.03	1.48	2.41
Total protein, g L^−1^	76.07	6.27	63.57	86.89	77.10	−0.35	−0.67
Albumin, g L^−1^	35.90	2.65	25.79	39.55	36.26	−2.07	5.68
Globulin, g L^−1^	40.05	6.33	27.99	52.08	40.61	−0.23	−0.78
Total bilirubin, μmol L^−1^	3.98	4.63	0.29	22.48	2.14	2.57	6.98
Total bilirubin, ln(μmol L^−1^)	0.94	0.91	−1.24	3.11	0.76	0.35	0.24
Haptoglobin, g L^−1^	0.25	0.27	0.03	0.91	0.10	1.46	0.69
Haptoglobin, ln(g L^−1^)	−1.91	0.98	−3.51	−0.09	−2.30	0.60	−0.87
AST^e^, U L^−1^	106.67	69.78	49.98	394.07	89.35	3.15	9.75
AST^e^, ln(U L^−1^)	4.56	0.42	3.91	5.98	4.49	1.90	4.32
GGT^f^, U L^−1^	28.78	9.66	16.27	63.94	26.63	1.50	3.64
GGT^f^, ln(U L^−1^)	3.32	0.31	2.79	4.16	3.28	0.40	0.20
AP^g^, U L^−1^	50.85	22.75	21.44	103.73	42.84	0.87	−0.21
AP^g^, ln(U L^−1^)	3.84	0.43	3.07	4.64	3.76	0.23	−0.88

^a^Skewness

^b^Kurtosis

^c^Non esterified fatty acids

^d^β-OH-butyric acid

^e^Aspartate amino transferase

^f^γ-glutamyl transferase

^g^Alkaline phosphatase

The greatest variability was observed for haptoglobin (CV of 108.0 %), total bilirubin (CV of 116.3 %) and NEFA (87.9 %). These variables were characterized by an asymmetrical distribution, with high skewness values, indicating that the tail on the right side was longer or fatter than the left side. These variables were also processed after natural logarithm transformation, with only a slight reduction of their variability.

The plasma parameters of energy metabolism, apart from the NEFA, were characterized by high variability with high CV and/or high range. The CV of glucose was not high (16.7 %), but the range was very wide (range of 4.54 mmol L^−1^), with a high kurtosis value (5.6), indicating that more of the variance is the result of infrequent extreme deviations (leptokurtic distributions). Conversely, total cholesterol was characterized by a high CV (47.3 %), in tune with its wide range (range of 5.1 mmol L^−1^) and a slight negative kurtosis (−1.57). BHBA was characterized by an intermediate CV compared with those of glucose and total cholesterol. For this ketone body the maximum value observed cannot be considered among the high values.

Plasma total protein and albumin were characterized by a low CV (8.2 and 7.4 %, respectively). The variability of globulin (CV of 15.8 %) was only slightly greater. Besides the low variability of these three variables, their range could be considered wide. For albumin only, the wide range was observed together with a high kurtosis (5.68), indicating that more of the variance is the result of infrequent extreme deviations.

Enzymes activities were characterized by high variability, with positive skewness. The natural logarithm transformation decreased both the skewness and their variability.

Table 3 shows the statistics of the prediction models based on FT-MIR spectroscopy for each plasma variable. The number of extracted factors with the PLS procedure for each prediction model ranged between 3 and 7. A low number of extracted factors was observed for some minerals (electrolytes, Ca, and Zn).

**Table 3:** Calibration curves and cross validation

Item	Extracted factors^a^	R^2^	RMSECV^b^	RER^c^	RPD^d^	S_r_^e^	RSD_r_^f^
Glucose, mmol L^−1^	5	0.66	0.3893	11.7	1.7	0.1550	3.86
Total cholesterol, mmol L^−1^	7	0.99	0.1156	43.8	13.8	0.0690	2.06
NEFA^g^, mmol L^−1^	6	0.86	0.1094	10.2	2.7	0.0325	9.80
NEFA^g^, ln(mmol L^−1^)	5	0.78	0.4414	6. 8	2.1	0.1214	7.99
BHBA^h^, mmol L^−1^	5	0.38	0.1012	5.6	1.3	0.0245	4.98
BHBA^h^, ln(mmol L^−1^)	7	0.60	0.1572	7.1	1.6	0.0669	9.03
Urea, mmol L^−1^	6	0.90	0.4623	13.0	3.1	0.2454	5.83
Creatinine, μmol L^−1^	4	0.60	6.7608	6.3	1.6	0.3080	0.31
Ca, mmol L^−1^	3	0.60	0.0791	6.3	1.6	0.0048	0.19
Inorganig P, mmol L^−1^	5	0.55	0.2336	7.3	1.5	0.0545	3.00
Mg, mmol L^−1^	4	0.55	0.0701	7.8	1.5	0.0063	0.62
Na, mmol L^−1^	4	0.57	2.2673	5.7	1.5	0.3289	0.23
K, mmol L^−1^	2	0.10	0.1920	5.1	1.0	0.0043	0.11
Cl, mmol L^−1^	6	0.70	1.3219	8.1	1.9	0.3198	0.30
Zn, μmol L^−1^	4	0.49	1.9577	5.6	1.4	0.2240	1.96
Ceruloplasmin, μmol L^−1^	3	0.59	0.6575	7.1	1.5	0.0705	2.17
Total protein, g L^−1^	5	0.99	0.7452	31.3	8.4	0.1561	0.21
Albumin, g L^−1^	6	0.96	0.5082	27.1	5.2	0.1362	0.38
Globulin, g L^−1^	4	0.98	0.8128	29.6	7.8	0.2665	0.67
Total bilirubin, μmol L^−1^	5	0.75	2.2967	9.7	2.0	0.6654	16.71
Total bilirubin, ln(μmol L^−1^)	3	0.66	0.5320	8. 2	1.7	0.0225	2.40
Haptoglobin, g L^−1^	4	0.62	0.1678	5.2	1.6	0.0148	5.97
Haptoglobin, ln(g L^−1^)	4	0.66	0.5734	6.0	1.7	0.0494	2.59
AST^i^, U L^−1^	6	0.69	39.5398	8.7	1.8	9.1112	8.54
AST^i^, ln(U L^−1^)	4	0.54	0.2856	7.2	1.5	0.0282	0.62
GGT^l^, U L^−1^	4	0.40	7.5093	6.3	1.3	0.3565	1.27
GGT^l^, ln(U L^−1^)	3	0.42	0.2344	5.8	1.3	0.0154	0.46
AP^m^, U L^−1^	7	0.66	13.2962	6.2	1.7	8.3024	16.33
AP^m^, ln(U L^−1^)	7	0.64	0.2614	6.02	1.6	0.1696	4.42

^a^Number of extracted factors with the PLS procedure

^b^Root mean square error of cross validation

^c^Range error ratio, obtained dividing the range of each variable by its standard deviation of prediction errors

^d^Ratio of prediction to deviation, obtained dividing the standard deviation of each variable with its standard deviation of prediction errors

^e^Repeatability of the standard deviation

^f^Repeatability relative standard deviation

^g^Non esterified fatty acids

^h^β-OH-butyric acid

^i^Aspartate amino transferase

^l^γ-glutamyl transferase

^m^Alkaline phosphatase

**Fig. 1:**
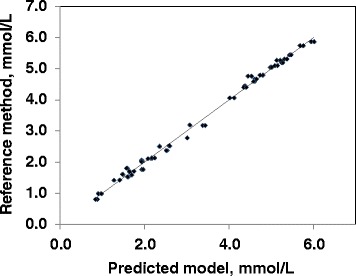
Linear regression plots for total cholesterol. Relationship between total cholesterol concentration (mmol L^−1^) measured by the reference method and by FT-MIR spectrometry in plasma using mid-infrared spectra (70 observations). (R2 = 0.995; root mean square error of cross validation = 0.1156; RER = 43.8; RER is the range error ratio, and is obtained dividing the range of total cholesterol with its standard deviation of prediction errors; RPD = 13.8, where RPD is the ratio of prediction to deviation, and is obtained dividing the standard deviation of total cholesterol with its standard deviation of prediction errors)

**Fig. 2:**
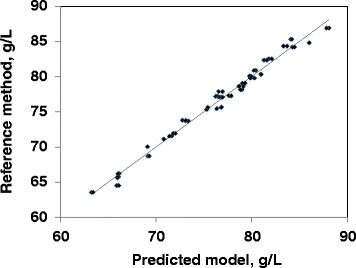
Linear regression plots for total protein. Relationship between total protein concentration (g L^−1^) measured by the reference method and by FT-MIR spectrometry in plasma using mid-infrared spectra (70 observations). (R2 = 0.985; root mean square error of cross validation = 0.7452; RER = 31.3; RER is the range error ratio, and is obtained dividing the range of total protein by its standard deviation of prediction errors; RPD = 8.4; RPD is the ratio of prediction to deviation, and is obtained dividing the standard deviation of total protein by its standard deviation of prediction errors)

The coefficient of determination (R^2^) of the prediction models ranged between 0.1 to values close to 1. Values greater than 0.9 were observed, in decreasing order, for the prediction models of total cholesterol (Fig. 1), total protein (Fig. 2), globulin, and albumin. The R^2^ values ranged between 0.75 and 0.9 for the prediction model of urea, NEFA, and total bilirubin. Values of R^2^ lower than 0.6 were observed for all minerals, with a slightly greater value only for Cl. The prediction models of enzymes activity were also characterized by low R^2^ values, ranging between 0.39 and 0.66.

The range error ratio (RER) and ratio to deviation (RPD) ranged between 5.1 and 43.8 for the former, and between 1 and 13.8 for the latter. Values of RPD greater than 5 were observed for total cholesterol, total protein, albumin, and globulin. RPD ranged between 2 and 5 for the prediction models of urea, NEFA, and total bilirubin. In general RPD and RER were low for minerals and enzymes activities.

The repeatability (S_r_) between the two values obtained with the two replicates of infrared spectra was good for almost all parameters considered in this study. Poor repeatability was observed for NEFA, BHBA, total bilirubin, AST and AP.

## Discussion

This study presented results concerning the use FT-MIR spectroscopy to predict plasma parameters in the metabolic profile of farm animals. In order to build a prediction model that accurately predicts unknown samples, the calibration data set should contain samples that represent all the possible sources of variation encountered when the prediction model will be used on an unknown sample. For this reason, the selection of samples is very important to provide the largest range of information for the building of the calibration data set [14]. The dairy cows selected for this study were all clinically healthy, consequently the variability observed through the data is representative of a normal physiological condition. Although the cows were not affected by clinical disease, the subjects of a population are often characterized by the presence of high variability for many blood biomarkers, and also for the occurrence of sub-clinical conditions [25]. In agreement with this consideration, all the concentrations of blood parameters measured in the present study were within the reference ranges [6, 26] reported for dairy cows without clinical signs of disease. To better represent the variability of blood biomarkers, we took blood samples of various dairy cow physiological stages, and reserved the transition period as having most relevant importance (14 of 35 samples). We made this choice because the peripartum period is the most critical phase for dairy cows. Important physiological, metabolic, and nutritional changes occur during transition [27, 28], and consequently, blood indices are subjected to marked changes [29–31]. In particular, parameters of energy and protein metabolism are strongly affected, as are glucose, NEFA, BHBA, urea and creatinine, as well as parameters related to an inflammatory response, such as haptoglobin, ceruloplasmin, total bilirubin, albumin, paraoxonase and cholesterol [25, 32, 33].

This study utilized a low number of blood samples, and the sampling was mainly focused around calving and limited to only one herd. The low number of samples used in this research suggested use of internal validation involving validation of the calibration models using the same sample set as that used for calibration development. Cross validation is a very reliable method of validation; it seeks to validate the calibration model on an independent test data set, but it does not use samples for testing only. These findings need to be confirmed with a further study on a wider population belonging to more herds, and with external validation with an independent and representative set of test objects, in order to give relevant and reliable estimates on the prediction ability of the predictive models.

The accuracy of infrared analysis is affected by the quality of the reference assays used [34]. In our study the reference assays used to analyze some blood parameters (i.e., BHBA, creatinine, ceruloplasmin, total bilirubin, haptoglobin, and GGT) were characterized by not having optimal repeatability between runs, with a CV greater than 3 %. For these blood parameters the developed calibration curves where characterized by a RPD lower than 2, which is considered to be the minimal threshold for acceptable approximate quantitative predictions. Among these blood variables without optimal repeatability in the reference chemistry, only the calibration curve of total bilirubin could be considered near to acceptability. Our results suggest that an improvement in the reference assays for all these blood parameters without optimal repeatability could improve the predictive ability of the calibration curve based on FT-MIR spectroscopy.

Poor prediction ability characterized the calibration curves of all minerals analyzed in this study. A similar result has been described in milk, mainly for electrolytes. In particular, Mg, Na and K showed poor prediction ability in FT-MIR spectroscopy [35]. Conversely, Soyeurt et al. [35] and Toffanin et al. [36] have suggested the potential of FT-MIR spectroscopy to predict Ca and P content in milk. Because the correlations between Ca and P concentrations estimated by the FT-MIR predictions and the known milk components were inferior to the correlation calculated based on the cross-validation, Soyeurt et al. [35] concluded that these equations of calibration were obtained from a real spectral absorbance. On the whole, these results confirm the difficulty in predicting mineral content in plasma with FT-MIR spectroscopy, in particular for minerals that are not included in organic compounds. Nevertheless, a similar condition also occurs for milk Ca, mainly included in casein under an organic (20 %) or inorganic form (46 % as tri-calcium phosphate) [37]. The difficulty observed in our study to predict mineral content in blood is likely due to two main reasons. First, among the minerals measured in the blood, a large proportion is in ionized form and not included in organic compounds. About 50 % of total Ca in plasma is in the ionized form and about 45 % is linked to protein, whereas, the quota of Mg ionized is about the 70 % [19]. Second, the variability “captured” for the mineral from the samples in this dataset was lower compared to variability measured for other blood parameters. A low variability, in particular for electrolytes, was observed. A better assay of minerals requires a dataset containing samples with a greater variability, which allows for an improvement in the predictive ability of FT-MIR spectroscopy. To increase the variability of these indices, it would be useful to include some animals with clinical diseases in the population.

A poor predictive ability characterized the calibration curves of all enzymes activities measured in this study. The inadequate estimation was probably due to the poor variability of these indices and also the non-normal distribution of the data, with a very low proportion of high values in this dataset. The log transformation did not provide an improvement in the predictive ability of the calibration curve. For a better assessment of enzymes activities, it is necessary to obtain a wider distribution of the data, in particular a representative number of samples with high values could improve the prediction ability of FT-MIR spectroscopy. An improvement is probable, considering that with FT-MIR spectroscopy, some authors were able to measure plasminogen concentration in milk and the distinction between plasmin and plasminogen [38], as well as secondary and tertiary structural changes in bovine plasminogen [39].

An excellent prediction ability was obtained for several of the parameters, mainly regarding energy, protein metabolism and inflammatory status. These results are very promising, but further studies are necessary to confirm the results and require validation on a larger population, data on different breeds, and herds raised in different environments. Among energy parameters, an excellent calibration curve was developed for total cholesterol. Interestingly, the determination of cholesterol in dairy products by using FT-MIR spectroscopy also showed good results [40].

Among the parameters of protein metabolism excellent calibration curves were developed for total protein, albumin and globulin. From this point of view, the FT-MIR technique is a widely used tool in many different fields and is even used to evaluate parameters with a “complex etiology” like secondary and tertiary protein structure studies [41, 42], differentiation of plasmin and plasminogen in milk [38], and determination of secondary and tertiary structural changes in bovine plasminogen [39] and casein [40].

For inflammatory status, it appears possible to predict with FT-MIR spectroscopy the blood parameters used to calculate the liver functionality index (LFI) according to Bertoni and Trevisi [4]. LFI measures the variation of some negative acute-phase proteins, which are reduced during inflammation, or related parameters (albumin, cholesterol, and total bilirubin) to help evaluate changes in liver function caused by inflammatory events. LFI takes into account changes in albumin, lipoproteins (measured as total cholesterol) and total bilirubin (its secretory enzymes are synthesized by the liver) occurring between 3 and 28 days in milk [2]. The lower the value of LFI, the more severe are the consequences of the inflammatory events that occur in the transition period and the inability of cows to adapt their metabolism to these challenges [31], causing a worsening of health status (clinical or subclinical problems). Our results may open an interesting perspective for an easier and more cost-effective approach to monitoring farm animals during critical periods, such as in the transition phase of dairy cows.

## Conclusions

Although the results of this study require further validation, they are very promising. We have shown that FT-MIR spectroscopy offers fairly accurate measurement of various plasma biomarkers of great importance for the evaluation of the metabolism and inflammatory status of dairy cows. All the animals selected in this investigation were healthy (i.e., without clinical events of disease) and the variability of the range of the checked parameters was restricted to a normal physiological condition. In these circumstances the accuracy required for the estimation of the biomarkers is very high and was reached for only some parameters. As such, FT-MIR technology may have even more success for the study of animals with pathological conditions (clinical and subclinical), especially when blood is used as main substrate for disease diagnosis. In this context, pathological changes occur for many markers and, in these conditions, it is certainly acceptable to have a greater range of error from an indirect assessment method with IR.

## Abbreviations

RPD: ratio to deviation
RER: ranger error ratio
NEFA: non esterified fatty acids
BHBA: β-OH-butyric acid
AST: aspartate amino transferase
GGT: γ-glutamyl transferase
AP: alkaline phosphatase
